# Nurse-led aerobic pulmonary rehabilitation: a comprehensive evaluation of physiological indicators, exercise tolerance, and quality of life in patients with moderate to severe COPD

**DOI:** 10.3389/fmed.2026.1828072

**Published:** 2026-04-30

**Authors:** Minyan Wang, Wenxian Xu, Cong Wang, Li Ye, Yajun Wang

**Affiliations:** 1 Department of Pulmonary and Critical Care Medicine, Quzhou People's Hospital, Quzhou, Zhejiang, China; 2 Department of Nursing, Quzhou People's Hospital, Quzhou, Zhejiang, China; 3 Department of Emergency, Quzhou People's Hospital, Quzhou, Zhejiang, China

**Keywords:** aerobic pulmonary rehabilitation, chronic obstructive pulmonary disease, exercise tolerance, nurse-led, physiological indicators, quality of life

## Abstract

**Background:**

Patients with moderate-to-severe chronic obstructive pulmonary disease (COPD) have difficulty performing pulmonary rehabilitation independently. In China, resources for respiratory physicians and rehabilitation specialists are limited and cannot meet the needs of all patients. The nurse-led model, leveraging the advantages of frequent nurse–patient contact, comprehensive case management capabilities, and relatively abundant resources, can help compensate for these deficiencies.

**Objective:**

To investigate the effects of a nurse-led aerobic pulmonary rehabilitation model on exercise tolerance, quality of life, and physiological status in patients with moderate to severe COPD.

**Methods:**

This study prospectively enrolled 80 patients with moderate to severe stable COPD who were treated at our hospital from January 2024 to August 2025 and were part of the intention-to-treat (ITT) population. They were randomly divided into an intervention group (*n* = 40) and a control group (*n* = 40). The control group received routine aerobic pulmonary rehabilitation nursing, while the intervention group received nurse-led multidisciplinary collaborative pulmonary rehabilitation intervention in addition to routine care. After 4 weeks, the per-protocol (PP) population totaled 74 cases (37 in each group). The primary outcome, the 6-min walk distance (6MWD), was compared between the ITT and PP populations. Secondary outcomes were assessed in the PP population, including the chronic obstructive pulmonary disease assessment test (CAT), St. George’s respiratory questionnaire (SGRQ), Barthel index (BI), and Borg scale scores.

**Results:**

Analysis of the primary outcome (6MWD) showed a statistically significant group-by-time interaction effect between the two groups in the ITT population (*p* < 0.05). At baseline, the 6MWD was 368.44 ± 45.07 m in the control group and 354.47 ± 56.17 m in the intervention group (*p* = 0.224). At 4 weeks, the 6MWD was 380.03 ± 44.70 m in the control group and 406.42 ± 34.01 m in the intervention group (*p* = 0.004), and the 6MWD in the intervention group was significantly higher than that before the intervention (*p* < 0.05). Consistent results were observed in the PP population (intervention group vs. control group: 405.03 ± 34.90 m vs. 378.06 ± 45.83 m, *p* = 0.006). Secondary outcomes were assessed only in the PP population. For CAT scores, baseline values were 12.90 ± 4.23 in the control group and 13.55 ± 3.81 in the intervention group (*p* = 0.488). At 4 weeks, scores decreased to 11.10 ± 2.72 and 9.15 ± 1.78 (*p* < 0.001). Both groups showed significant within-group improvements (*p* < 0.05). For SGRQ scores, no significant between-group difference was found (control: 56.80 ± 9.76 vs. intervention: 54.23 ± 7.02, *p* = 0.196), but intra-group comparison showed significant improvement in the intervention group compared to baseline (*p* < 0.05). For Borg scores, the intervention group scored significantly lower than the control group (3.26 ± 0.27 vs. 4.10 ± 0.46, *p* < 0.001). For BI, the intervention group scored significantly higher than the control group (64.22 ± 6.30 vs. 56.49 ± 4.40, *p* < 0.001). All secondary outcomes showed statistically significant group-by-time interaction effects (*p* < 0.05).

**Conclusion:**

The nurse-led aerobic pulmonary rehabilitation model can effectively improve exercise tolerance, respiratory status, and activities of daily living in patients with moderate to severe COPD. However, the long-term efficacy of nurse-led pulmonary rehabilitation and patient treatment adherence still require further evaluation.

## Introduction

Chronic obstructive pulmonary disease (COPD) is a progressive yet preventable chronic condition. According to the global burden of disease study (GBD), the number of COPD cases worldwide reached 212 million in 2019, with an age-standardized prevalence of 3,286.2 per 100,000 population, representing an increase of approximately 25% over the past three decades (1). With its rising incidence year by year, it has become a global health issue and is the third leading cause of death worldwide (2). COPD is caused by the inhalation of harmful particles, particularly from smoking and air pollution (3). Its main features include coughing or wheezing, excessive sputum production accompanied by dyspnea, which may further lead to deterioration of cardiopulmonary function (1). Assessments indicate that the prevalence of COPD increases exponentially with age, the prevalence rate is as high as 9–10% among people over 40 years of age, and rises to over 15% among those aged 60 and above (4). Globally, approximately 40 million people aged 60 years and older suffer from COPD annually, commonly with multiple comorbidities (5). The disease burden is particularly severe in patients with moderate-to-severe COPD. Compared to those with mild-to-moderate COPD, patients with moderate-to-severe COPD have severely impaired lung function and often present with persistent dyspnea, respiratory muscle fatigue, exercise-induced hypoxemia, as well as an increased risk of falls and arrhythmias (4). These patients experience a marked decline in exercise tolerance, worsened quality of life, and a higher frequency of acute exacerbations, leading to repeated hospitalizations and substantial consumption of healthcare resources (6). Moderate-to-severe COPD not only carries a high risk of comorbidities but is also a primary reason for increased hospitalization rates and rising home healthcare costs (7).

Currently, clinical treatment for COPD primarily involves pharmacotherapy and oxygen therapy (8). Pulmonary rehabilitation has been proven as an effective non-pharmacological intervention that significantly improves exercise tolerance, dyspnea, and quality of life in patients with COPD. It is strongly recommended by international guidelines as one of the core management strategies for COPD (9). However, for patients with moderate to severe COPD, the implementation of pulmonary rehabilitation presents unique challenges. These patients often exhibit persistent dyspnea, respiratory muscle fatigue, exercise-induced hypoxemia, and an increased risk of falls and arrhythmias, making it difficult for them to perform rehabilitation exercises safely and effectively on their own (4, 6). In China, with the exception of first-tier cities, there is a widespread shortage of respiratory physicians and rehabilitation specialists, and their opportunities to lead pulmonary rehabilitation programs are very limited. The shortage of rehabilitation specialists in China is a core bottleneck restricting the implementation of pulmonary rehabilitation. Under the traditional model, pulmonary rehabilitation therapy heavily relies on respiratory physicians and rehabilitation specialists to provide one-on-one exercise guidance and supervision (10). The number of respiratory physicians and rehabilitation specialists in China falls far short of clinical demand, resulting in the vast majority of patients with moderate-to-severe COPD being unable to receive timely and continuous rehabilitation services. This highlights the urgent need to explore alternative models of pulmonary rehabilitation services (5). Moreover, such models typically require patients to have a relatively stable functional status and access to a fixed training facility, making them ill-suited to accommodate the significant symptom fluctuations and high rehabilitation risks characteristic of patients with moderate to severe COPD (11). Compared with respiratory physicians, nurses have more frequent contact with patients before and after surgery, enabling them to conduct comprehensive assessments and make dynamic adjustments during the implementation of pulmonary rehabilitation strategies (12). In this context, the nurse-led pulmonary rehabilitation model demonstrates unique clinical value. Nurses possess advantages such as the ability to provide continuous dynamic monitoring and risk warning, deep integration of symptom management with rehabilitation training, and the promotion of adherence through psychological and behavioral interventions (13). Patients with moderate to severe COPD, who repeatedly experience severe exercise-induced dyspnea, are prone to fear, avoidance behaviors, and decreased self-efficacy. By establishing long-term trusting relationships and frequent interactions, nurses can effectively implement motivational interviewing, peer support, and behavioral incentives, thereby significantly improving patients’ adherence to rehabilitation (14). Preliminary evidence has demonstrated the feasibility and potential value of nurse-led pulmonary rehabilitation. For instance, Zhang et al. showed that well-trained nurses can safely and effectively deliver a simplified pulmonary rehabilitation protocol, improving exercise capacity and quality of life in rural COPD patients (15). Zakrisson et al. (16) reported that a nurse-led multidisciplinary pulmonary rehabilitation program at the primary care level reduced dyspnea symptoms and improved certain quality of life indicators, with benefits lasting up to 3 years. However, most of these studies have focused on patients with mild-to-moderate COPD or highly simplified protocols, and there remains a lack of high-quality evidence for a nurse-led pulmonary rehabilitation model that targets moderate-to-severe COPD, integrates the role of an alternative physiotherapist, and is adaptable to low-resource settings.

Therefore, based on previous work, relevant guidelines, and clinical experience, this study explored a nurse-led aerobic pulmonary rehabilitation strategy for patients with moderate to severe COPD. The primary research question is: In patients with moderate to severe COPD, does a nurse-led aerobic pulmonary rehabilitation model, compared with routine aerobic pulmonary rehabilitation nursing, improve exercise tolerance, quality of life, and physiological status? This study aims to provide an evidence-based foundation for optimizing rehabilitation nursing protocols for this patient population.

## Materials and methods

### Study subjects

Patients with acute moderate to severe COPD who were treated at Quzhou People’s Hospital from January 2024 to August 2025 were prospectively enrolled. Inclusion criteria: (1) Meet the GOLD guidelines for the diagnosis of stable moderate-to-severe COPD (13), with a forced expiratory volume in the first second/forced vital capacity (FEV_1_/FVC) < 0.70 for confirmation, and FEV_1_% predicted between 30 and 80% to determine moderate-to-severe severity; (2) Age ≥18 years; (3) Patients able to cooperate with pulmonary function tests; (4) Adequate oxygenation: Oxygenation index >150 mmHg with inhaled oxygen concentration ≤40% and positive end-expiratory pressure ≤5 cm H_2_O; (5) Hemodynamically stable; (6) Voluntary participation in this study and signing of informed consent. Exclusion criteria: (1) Mental or intellectual disabilities preventing normal communication; (2) Liver or kidney dysfunction; (3) Concurrent malignant tumors or end-stage disease (expected survival time <7 days); (4) Other respiratory diseases; (5) Poor compliance. Dropout criteria: (1) Inability to continue the intervention due to time conflicts caused by family emergencies; (2) Loss of contact due to changes in contact information. Based on *α* = 0.05, 1 − *β* = 0.90, with the change in 6-min walk distance (6MWD) before and after the intervention as the main effect, the minimum detectable difference was set at 50 m with a standard deviation of 10. The minimum required sample size was calculated using PASS 15.0 software, indicating at least 29 patients per group, totaling 58 patients. Considering a 20% dropout rate, the minimum sample size required was 70 patients, with 35 patients in each group. This trial was conducted in accordance with the Declaration of Helsinki. Written informed consent was obtained from all patients, and the study was approved by the Ethics Committee of Quzhou People’s Hospital (Approval No.: 2023–104). This study has been approved by the Chinese Clinical Trial Registry (Registration Number: ChiCTR2500093179).

### Grouping and blinding methods

**Figure 1:**
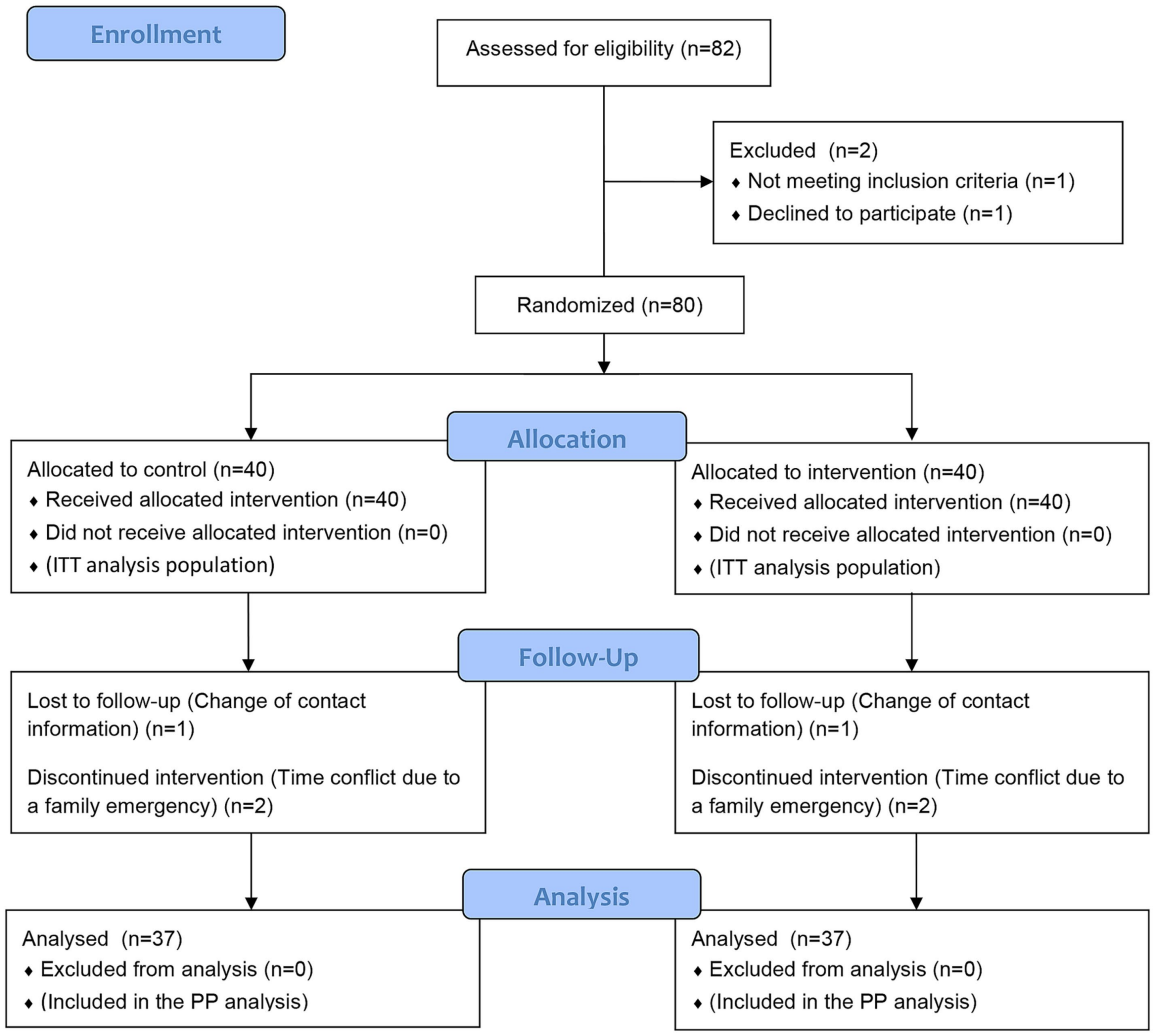
Patient inclusion and exclusion flowchart.

A total of 80 patients were enrolled in the intention-to-treat (ITT) analysis and were divided into an intervention group (*n* = 40) and a control group (*n* = 40) using a random number table. First, a statistician who was not involved in clinical care or outcome assessment generated a 1:1 random allocation sequence using SPSS software and produced the corresponding random number table based on this sequence. A total of 80 consecutively numbered patients (numbered 1 to 80) were assigned to either the intervention group or the control group according to the random number table sequence, with odd numbers assigned to the control group and even numbers to the intervention group (40 patients per group). The allocation scheme was sealed in opaque envelopes and kept by an independent research coordinator. Once patients met the inclusion criteria and signed informed consent forms, the coordinator opened the envelopes sequentially and assigned the patients to their respective groups. The entire process of enrollment, grouping, and allocation was carried out by this coordinator, and neither the clinical nurses nor the outcome assessors participated in the grouping process. To ensure a balanced number of cases between the two groups, patients with odd numbers were assigned to the control group, and those with even numbers were assigned to the intervention group. The control group received routine aerobic pulmonary rehabilitation nursing, while the intervention group received a nurse-led aerobic pulmonary rehabilitation intervention in addition to routine care. During the study period, 6 patients dropped out, and 74 patients ultimately completed the intervention, comprising the per-protocol (PP) population, with 37 patients in the intervention group and 37 patients in the control group (Figure 1).

This study adopted an open-label design. Due to the nature of the intervention, blinding of participants and the nurses administering the intervention was not feasible. To minimize measurement bias, all outcome assessors and statistical data analysts were kept blinded to group allocation. Statistical analysis was conducted using coded grouping, and the blinding was not broken until the analysis was completed.

### Nursing interventions for the control group

Within 24 h of admission, the control group received health education, which provided patients with fundamental knowledge related to COPD to help them understand the etiology of the disease. Training and education were provided to help patients understand their symptoms and management methods, and the importance of pulmonary rehabilitation exercises was explained. Subsequently, they received a one-week inpatient routine airway care and early rehabilitation exercise program, including assisting patients with turning over, nebulized inhalation, back percussion for sputum expulsion, sputum drainage, and sequential treatment nursing of invasive mechanical ventilation-non-invasive mechanical ventilation-high-flow nasal cannula oxygen therapy. Patients were assisted with limb activities, including the implementation of limb compression therapy, ankle pump exercises, and practice on treadmills and exercise bikes. Medication guidance was provided, assisting patients in taking medications on time and in the correct dosage as prescribed by the respiratory physician, to control disease progression and alleviate symptoms. Dietary guidance was also provided, recommending a high-protein, low-salt, low-fat diet, focusing on a balanced diet to ensure adequate calorie and nutrient intake. During hospitalization, patient status was assessed daily, and body weight changes were monitored. On the seventh day of hospitalization, a COPD health education booklet was distributed to avoid triggers, and the pulmonary rehabilitation video was copied for patients to study the relevant content and implement it. During the 3 weeks after patient discharge, telephone follow-ups were conducted every 3 days to inquire about the patient’s general condition and the implementation of pulmonary rehabilitation exercises, collect data, and answer patient questions. A home follow-up was conducted 3 weeks after discharge, lasting approximately 1 h, to collect data on self-efficacy, self-management ability, degree of dyspnea, pulmonary function, and quality of life, and to answer patient questions. The nursing course of the program was 4 weeks.

### Nursing interventions for the intervention group

The intervention group, in addition to the interventions provided to the control group, implemented a pulmonary rehabilitation program led by nurses. This program was developed and validated jointly by three nurses, one respiratory physician, and one rehabilitation specialist. The respiratory physician is responsible for medical decision-making, including the development and adjustment of sequential respiratory support protocols and overall safety supervision during rehabilitation. The rehabilitation specialist focuses on the technical aspects of rehabilitation, including the development of exercise prescriptions and training nurses to perform various rehabilitation techniques. Under the supervision of the respiratory physician and the technical guidance of the rehabilitation specialist, the nurses are responsible for the daily implementation of rehabilitation training, dynamic monitoring, patient education, psychological support, and data collection. The teaching sessions in this study were conducted by nurses with the assistance of a rehabilitation specialist and a respiratory physician. All teaching sessions used standardized slide presentations and video materials to ensure consistency and reproducibility of the educational content. The nurse-led pulmonary rehabilitation intervention includes the following core components, which are independently performed by nurses without real-time involvement of physiotherapists or respiratory physicians: (1) daily monitoring of vital signs and oxygen saturation; (2) guided breathing function training; (3) airway clearance techniques; (4) implementation of progressive exercise training; (5) precision fluid management; (6) aspiration prevention guidance during oral feeding and medication administration; (7) motivational psychological support and patient education; (8) telephone follow-ups and home visits during the home-based rehabilitation period. The following components require decision-making or supervision by the rehabilitation specialist or respiratory physician: (1) initial development and periodic adjustment of exercise prescriptions; (2) development and adjustment of sequential respiratory support protocols; (3) adjustment of treatment plans when adverse events occur during rehabilitation. Before the intervention, the three nurses received a 2-week specialized training program delivered jointly by the rehabilitation specialist and the respiratory physician. The training included: (1) theoretical lectures covering the basic pathophysiology of COPD, principles of pulmonary rehabilitation, interpretation of exercise prescriptions, and risk identification and management procedures; (2) skills training covering breathing function training techniques, airway clearance techniques, implementation and intensity adjustment of progressive exercise training, aspiration prevention techniques, and precision fluid management; (3) clinical practice, during which nurses performed the full rehabilitation protocol on five simulated patients under the supervision of the rehabilitation specialist and respiratory physician, and were required to pass assessments before independent implementation. After training, nurses were required to pass both theoretical and practical competency assessments to qualify for independent intervention implementation. During the intervention period, the rehabilitation specialist conducted on-site spot checks of nurse performance once every 2 weeks to ensure quality of execution. Pulmonary rehabilitation nurses were responsible for the overall coordination, implementation, dynamic evaluation, and cross-team communication of the program, and utilized the hospital’s case system platform for comprehensive data collection and outcome tracking.

The nurse-led pulmonary rehabilitation intervention lasted 4 weeks, including 1 week of intensive health education and exercise/breathing guidance in the hospital, followed by 3 weeks of home-based breathing rehabilitation exercises and exercise training. Meanwhile, researchers provided guidance and conducted follow-ups with the subjects weekly via telephone.

Based on the primary risk factors in patients with moderate to severe COPD, including skeletal muscle atrophy, respiratory muscle fatigue, and impaired mucociliary clearance function, we specifically developed a guidance manual for these patients. This manual contains recommendations for active smoking cessation guidance, avoidance of passive smoking, improvement of airway management, and enhancement of exercise tolerance. The manual was distributed to patients within 24 h of admission. During the first week of hospitalization, patients received interventions focused on improved airway management, precision fluid management, progressive exercise training, oral feeding and medication administration, and motivational psychological support nursing. Detailed nursing operations performed in the hospital are shown in Table 1. In the first week, researchers conducted intensive on-site lectures for patients, including theoretical explanations and operational demonstrations. The teaching sessions were held in the health education classroom of the target hospital. Before the lectures, self-developed disease-related knowledge manuals were distributed to participants, and health education slideshows and videos were played centrally. After the theoretical lectures, on-site demonstrations were conducted, such as pursed-lip breathing techniques and correct effective coughing methods. Participants and their family members received education together. Each teaching session was controlled within 30 to 45 min. After teaching, participants engaged in exchanges and discussions with researchers to better master the health education content. Researchers conducted two intensive lectures for patients during the first week, and all health education was completed within 1 week. During hospitalization, nurses performed daily bedside assessments and recorded the results in the case management form, monitoring vital signs, oxygen saturation, degree of dyspnea, level of fatigue, symptom records, fluid intake and output, and exercise performance. The intensity, duration, and progression criteria of the rehabilitation training were dynamically adjusted based on the patient’s Borg score, oxygen saturation (SpO_2_), and subjective feelings of fatigue. On a weekly basis, the rehabilitation specialist determined whether to advance the intensity or duration for the following week according to the above criteria, taking into account the patient’s rehabilitation diary data, changes in 6MWD, and Borg score trends. All progression decisions were recorded in the case management form to ensure the traceability and reproducibility of the protocol.

**Table 1:** Nurse-led aerobic pulmonary rehabilitation procedures.

Intervention content		Methods	Frequency	Responsible professional
Improved airway management	Sequential respiratory support	Day 1, pressure-controlled ventilation (PCV) mode is used to adjust transpulmonary pressure. Day 2–3, ECMO support combined with high-flow oxygen therapy was used to regulate spontaneous breathing drive. Day 4–7, awake prone positioning ventilation	1–2 times daily for 2–3 h per session.	Nurse (with respiratory physician oversight)
	Guided breathing function training	Day 1–2, the focus was on breath control and re-establishing a normal breathing pattern. Day3-4, diaphragmatic resistance training was performed to improve lung function. Day 5–7, respiratory muscle resistance training.	4 times daily, 30 min per session.	Nurse (trained by rehabilitation specialist)
	Protective airway clearance	Perform the following sequence: 3–5 repetitions of breath control, 3–5 repetitions of thoracic expansion, 3–5 repetitions of breath control, 2–3 forced expirations, and 3–5 repetitions of breath control.	4 times daily, 20 cycles per session.	Nurse
Precision fluid management		Establishing precise volume targets Accurately recording 24-h intake and output Maintaining effective circulation	Record fluid imbalance status and make adjustments daily at 07:00, 15:00, and 22:00, as well as 30 min after diuretic administration.	Nurse (with respiratory physician oversight)
Progressive exercise training		Day 1, upright bedside training Day 2–3, controlled aerobic exercise on a stationary bicycle Day 4–7, upper and lower limb strength training.	4 times daily, 30 min per session.	Nurse (protocol developed by rehabilitation specialist)
Oral feeding and medication administration		Upon admission, patients were instructed to lower their head when swallowing during oral feeding and medication administration, supplemented by the Mendelsohn maneuver, to prevent aspiration.	Daily	Nurse
Motivational psychological support		Family-participated rehabilitation training Medical staff support Peer support	Daily	Nurse

**Figure 2:**
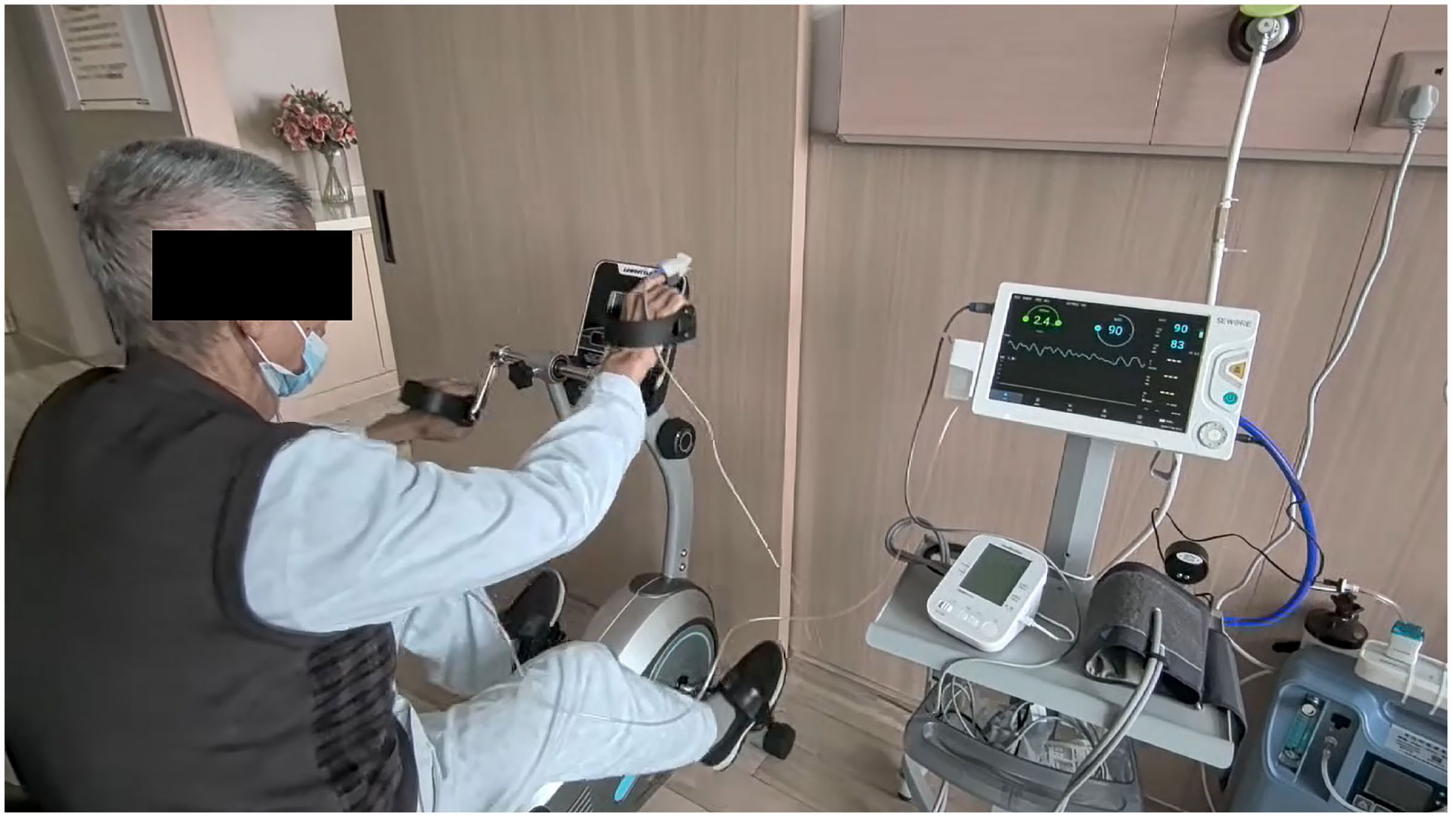
Nurse-led pulmonary rehabilitation training. Debugging constant-speed pedaling rehabilitation bicycle, equipped with heart rate and blood pressure monitoring devices, patients undergo progressive exercise training.

Starting from the second week, participants performed self-managed breathing rehabilitation at home and returned to the outpatient department daily to receive progressive exercise training (Figure 2). Meanwhile, after the initiation of home care, in addition to daily monitoring of exercise training, researchers conducted weekly telephone follow-ups to understand patients’ rehabilitation training progress, answer their questions, and properly supervise and guide their rehabilitation training. After discharge, patients in the intervention group received weekly telephone follow-ups, each lasting approximately 15–20 min. The telephone follow-ups were conducted by the nurse responsible for the patient, using a standardized telephone follow-up record form to collect information on the patient‘s rehabilitation training performance over the past week, degree of dyspnea and fatigue, oxygen saturation and heart rate, symptom changes, and adverse events. Participants who encountered problems during training could consult researchers by telephone. Home visits were conducted at the end of the second week and the end of the third week after discharge to inquire whether patients’ symptoms such as cough, sputum production, wheezing, and dyspnea had been alleviated over the past 3 weeks, and whether their quality of life had improved. Relevant indicators and goal completion status were collected to clarify patients’ self-management status at home. Each home follow-up visit lasted approximately 1 h. The detailed comparison between the control group and the intervention group is shown in Table 2.

**Table 2:** Comparison of nursing interventions between the control group and the intervention group.

Intervention component	Control group	Intervention group
Health education (COPD basic knowledge, symptom management, rehabilitation importance)	✓	✓
Routine airway care (turning over, nebulized inhalation, back percussion, sputum drainage)	✓	✓
Sequential respiratory support (invasive/non-invasive ventilation, high-flow oxygen)	✓	✓
Limb activities (compression therapy, ankle pump exercises)	✓	✓
Medication guidance	✓	✓
Dietary guidance (high-protein, low-salt, low-fat diet)	✓	✓
COPD health education booklet and rehabilitation video	✓	✓
Telephone follow-up (every 3 days post-discharge)	✓	✓
Home visit (at 3 weeks post-discharge)	✓	✓
Nurse-led multidisciplinary pulmonary rehabilitation program	—	✓
Pulmonary rehabilitation nurse as case manager (dynamic assessment, real-time exercise adjustment)	—	✓
Specialized nurse-led progressive exercise training (bed-based → sitting → standing → walking, with daily intensity adjustment)	—	✓
Guided breathing function training (diaphragmatic resistance, respiratory muscle training, 4 times daily)	—	✓
Protective airway clearance techniques (active cycle of breathing techniques)	—	✓
Precision fluid management (strict intake/output recording, daily adjustments)	—	✓
Oral feeding and medication administration with aspiration prevention (Mendelsohn maneuver)	—	✓
Motivational psychological support (family participation, peer support, daily encouragement)	—	✓
Intensive on-site lectures (theoretical + practical demonstrations in first week)	—	✓
Weekly telephone follow-up (in addition to routine calls) with progress tracking	—	✓
Additional home visits (at end of week 2 and week 3 post-discharge)	—	✓
Case system platform for comprehensive data collection and outcome tracking	—	✓

### Assessments and outcomes

#### Primary outcome

The primary efficacy endpoint was the 6MWD in patients with moderate to severe COPD assessed during the 4-week follow-up after nursing. Analysis of the primary efficacy endpoint was performed on both the ITT and PP populations. The 6MWD was chosen as the primary outcome because it is the gold standard measure of exercise tolerance in COPD, offering strong reproducibility, sensitivity to intervention effects, and significant prognostic value. 6MWD: The subject is required to walk back and forth along a flat, unobstructed, and well-ventilated corridor measuring 30 meters in length. Before the test, the subject needs to familiarize themselves with the testing method and environment and walk as quickly as possible. The pace may be adjusted if necessary, including slowing down or taking a brief rest. The researcher does not intervene with the subject until the 6-min test is completed. Prior to conducting the 6-min walk test, the researcher should ensure they are aware of the patient’s medical history and consider the precautions and contraindications for this test (15).

#### Secondary outcome

The secondary efficacy endpoints included the results of the chronic obstructive pulmonary disease assessment test (CAT), St. George’s respiratory questionnaire (SGRQ), Barthel Index (BI), and Borg score after 4 weeks of nursing. For secondary outcomes, the CAT and the SGRQ are internationally recognized COPD-specific quality-of-life instruments that have been widely used in pulmonary rehabilitation studies. The BI was selected to assess activities of daily living due to its simplicity, applicability to both inpatient and home-based rehabilitation settings, and good discriminative ability in COPD patients. The Borg score was chosen to evaluate dyspnea severity because it is simple, allows real-time assessment, and enables nurses to dynamically adjust training intensity during rehabilitation sessions. The secondary outcomes are exploratory in nature. The ITT population has a small sample size with a non-random dropout pattern, and imputing missing data could introduce substantial bias. Therefore, the secondary analyses were restricted to the PP population to obtain unbiased estimates among protocol adherers. The secondary efficacy endpoints were assessed in the PP population. CAT: This scale reflects the impact of the disease on patients’ physical, psychological, and daily life aspects (17). SGRQ: This scale comprises three dimensions: symptoms, activity, and impact on daily life, containing a total of 50 items. Each section is scored out of a total of 100 points; higher scores indicate poorer quality of life (17). BI: Used to assess the patient‘s level of fatigue, where a lower score indicates a higher degree of fatigue (18). Borg score: Used to assess the degree of dyspnea or fatigue. A higher score indicates more severe dyspnea (14). Daily training was dynamically adjusted by the nurse based on the patients’ Borg score, oxygen saturation, and subjective feelings. On a weekly basis, the rehabilitation specialist determined whether to advance the intensity or duration for the following week according to the above criteria, taking into account the patient’s rehabilitation diary data, changes in 6MWD, and Borg score trends. All progression decisions were recorded in the case management form to ensure the traceability and reproducibility of the protocol.

### Statistical analysis

Data analysis was performed using SPSS 27.0 software (IBM Corporation, Armonk, NY). All subjects who met the screening criteria and completed randomization were included in the ITT population. The PP population was defined as the group of patients who were randomized, received the treatment to which they were assigned, completed the 6-week assessment, and had no major protocol deviations. The primary efficacy analysis was performed on both the ITT and PP populations. The Shapiro–Wilk test was used to assess normality. Quantitative data with a normal distribution were expressed as mean ± standard deviation ( $\bar{x\;}\pm s$ ) and analyzed using an independent samples t-test for comparisons between two groups. Quantitative data with a non-normal distribution were expressed as median (first quartile, third quartile) [M (P_25_, P_75_)] and analyzed using the Mann–Whitney U test for comparisons between two groups. For normally distributed quantitative data, comparisons between two time points were performed using a paired samples t-test, and comparisons among multiple time points were performed using repeated measures ANOVA. For non-normally distributed quantitative data, comparisons among multiple time points were performed using the Friedman test. Categorical data were expressed as number of cases (percentage) [*n* (%)] and analyzed using the Chi-square (*χ*^2^) test. All *p*-values were two-tailed, and a *p* < 0.05 was considered statistically significant.

## Results

### Comparison of baseline characteristics between the two groups

There were no statistically significant differences in baseline characteristics such as age, gender, BMI, marital status, severity grade, course of disease, smoking, diabetes, and hypertension in the ITT population (*p* > 0.05) (Table 3).

**Table 3:** Baseline characteristics of all patients.

Characteristics	Control group (*n* = 40)	Intervention group (*n* = 40)	*t*/*χ*^2^	*p*
Age, y ( $\bar{x}\pm s$ )	68.68 ± 8.94	69.16 ± 7.58	0.259	0.796
Gender [*n* (%)]			0.213	0.644
Male	24 (60.00)	26 (65.00)		
Female	16 (40.00)	14 (35.00)		
BMI, kg/m^2^ ( $\bar{x}\pm s$ )	20.45 ± 2.16	21.11 ± 1.88	1.458	0.149
Marital status [*n* (%)]			0.738	0.390
With spouse	34 (85.00)	31 (77.50)		
Without spouse	6 (15.00)	9 (23.50)		
Severity grade [*n* (%)]			0.621	0.431
Moderate	29 (72.50)	32 (80.00)		
Severe	11 (27.50)	8 (20.00)		
Course of disease, y ( $\bar{x\;}\pm s$ )	4.26 ± 1.75	4.33 ± 1.51	0.192	0.849
Smoking /day [*n* (%)]			1.638	0.651
No	8 (20.00)	7 (17.50)		
1–10	27 (67.50)	29 (72.50)		
11–20	5 (12.50)	3 (7.50)		
21–40	0 (0.00)	1 (2.50)		
Diabetes [*n* (%)]			0.346	0.556
Yes	8 (20.00)	6 (15.00)		
No	32 (80.00)	34 (85.00)		
Hypertension [*n* (%)]			0.125	0.723
Yes	4 (10.00)	5 (12.50)		
No	36 (90.00)	35 (87.50)		

### Comparison of 6MWD between the two groups

Of the 80 patients, 74 completed the nursing care and follow-up according to the protocol. In the control group, 2 patients discontinued the intervention due to schedule conflicts and 1 was lost to follow-up, resulting in a total of 37 patients who completed the treatment and follow-up per protocol. In the intervention group, 2 patients discontinued the intervention due to schedule conflicts and 1 was lost to follow-up, resulting in a total of 37 patients who completed the treatment and follow-up per protocol. According to the ITT analysis, there was a statistically significant group-by-time interaction effect for 6MWD between the control group and the intervention group (*p* < 0.05). At the 4-week intervention, the difference between the two groups remained statistically significant (*p* < 0.05), and the 6MWD in the intervention group at 4 weeks was significantly higher than that before the intervention (*p* < 0.05). Consistent results were also observed in the PP population (Table 4).

**Table 4:** 6MWD of ITT and PP.

Characteristics	0 week	4 weeks	*Mauchly W*	*p*	*F*	*p*	Partial Eta squared
ITT-6MWD, m ( $\bar{x}\pm s$ )							
Control group (*n* = 40)	368.44 ± 45.07	380.03 ± 44.70	0.972	0.125	8.823	0.004	0.091
Intervention group (*n* = 40)	354.47 ± 56.17	406.42 ± 34.01^*^					
t	1.227	2.971					
*P*	0.224	0.004					
95% CI	−3.216 ~ 2.331	−5.892 ~ 2.477					
PP-6MWD, m ( $\bar{x}\pm s$ )							
Control group (*n* = 37)	368.13 ± 46.80	378.06 ± 45.83	0.986	0.131	8.195	0.005	0.084
Intervention group (*n* = 37)	353.19 ± 58.23	405.03 ± 34.90^*^					
t	1.216	2.849					
*P*	0.228	0.006					
95% CI	−8.094 ~ 3.831	−4.899 ~ 3.582					

^*^p < 0.05 compared with the value at 0 week.

### Assessment of quality of life between the two groups of patients

**Figure 3:**
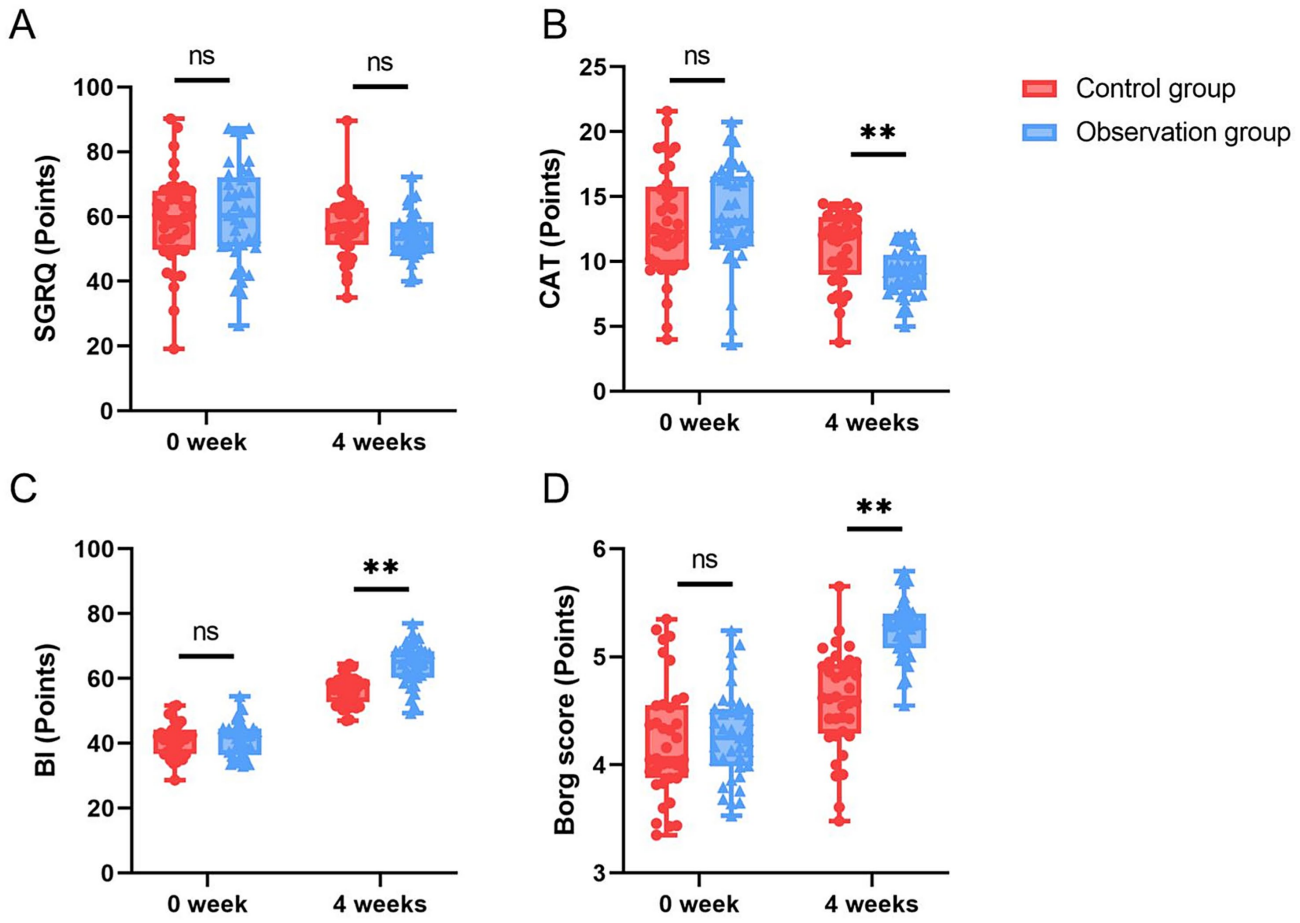
Secondary outcomes of PP. **(A)** SGRQ of the control group and the intervention group at week 0 and week 4. **(B)** CAT of the control group and the intervention group at week 0 and week 4. **(C)** BI of the control group and the intervention group at week 0 and week 4. **(D)** Borg score of the control group and the intervention group at week 0 and week 4.

Secondary efficacy endpoints were analyzed in the PP population. The difference in the group-by-time interaction effect for SGRQ between the two groups was not statistically significant, and the difference in the main effect of group between the two groups was not statistically significant either (*p* > 0.05). The SGRQ score in the intervention group at 4 weeks was significantly lower than that before the intervention (*p* < 0.05) (Table 5; Figure 3A).

**Table 5:** Quality of life of PP.

Characteristics	0 week	4 weeks	*Mauchly W*	*p*	*F*	*p*	Partial Eta squared
SGRQ, points ( $\bar{x}\pm s$ )							
Control group (*n* = 37)	58.84 ± 14.53	56.80 ± 9.76	1.000	0.947	0.895	0.347	0.022
Intervention group (*n* = 37)	60.26 ± 15.46	54.23 ± 7.02^*^					
*t*	0.406	1.304					
*p*	0.686	0.196					
95% CI	−3.831 ~ 4.402	−2.067 ~ 2.182					
CAT, points ( $\bar{x}\pm s$ )							
Control group (*n* = 37)	12.90 ± 4.23	11.10 ± 2.72^*^	0.943	0.121	5.997	0.017	0.073
Intervention group (*n* = 37)	13.55 ± 3.81	9.15 ± 1.78^*^					
*t*	0.697	3.654					
*p*	0.488	<0.001					
95% CI	−6.542 ~ 2.688	−2.157 ~ 2.496					

*p < 0.05 compared with the value at 0 week.

Analysis of the PP population showed that the group-by-time interaction effect for CAT between the two groups was statistically significant (*p* < 0.05). At 4 weeks, the difference in the main effect of group between the two groups was statistically significant, with the intervention group being significantly lower than the control group (*p* < 0.05). The CAT scores in both the control group and the intervention group at 4 weeks were significantly lower than those before the intervention (*p* < 0.05) (Table 5; Figure 3B).

### Assessment of physiological status between the two groups of patients

Secondary efficacy endpoints were analyzed in the PP population. The difference in the group-by-time interaction effect for BI between the two groups was statistically significant (*p* < 0.05). At 4 weeks, the difference in the main effect of group between the two groups was statistically significant, with the intervention group being significantly higher than the control group (*p* < 0.05). The BI scores in both the control group and the intervention group at 4 weeks were significantly higher than those before the intervention (*p* < 0.05) (Table 6; Figure 3C).

**Table 6:** Physiological status of PP.

Characteristics	0 week	4 weeks	*Mauchly W*	*p*	*F*	*p*	Partial Eta squared
BI, points ( $\bar{x}\pm s$ )							
Control group (*n* = 37)	41.23 ± 5.18	56.49 ± 4.40^*^	0.965	0.137	25.867	<0.001	0.144
Intervention group (*n* = 37)	41.25 ± 5.27	64.22 ± 6.30^*^					
t	0.017	6.122					
*p*	0.987	<0.001					
95%CI	−2.572 ~ 1.298	−6.033 ~ 1.087					
Borg score, points ( $\bar{x}\pm s$ )							
Control group (*n* = 37)	4.23 ± 0.54	4.10 ± 0.46^*^	1.000	0.962	18.395	<0.001	0.124
Intervention group (*n* = 37)	4.27 ± 0.42	3.26 ± 0.27^*^					
*t*	0.393	9.579					
*p*	0.695	<0.001					
95%CI	−4.402 ~ 3.329	−2.047 ~ 3.385					

*p < 0.05 compared with the value at 0 week.

Analysis of the PP population showed that the group-by-time interaction effect for the Borg score between the two groups was statistically significant (*p* < 0.05). At 4 weeks, the difference in the main effect of group between the two groups was statistically significant, with the intervention group being significantly lower than the control group (*p* < 0.05). The Borg scores in both the control group and the intervention group at 4 weeks were significantly lower than those before the intervention (*p* < 0.05) (Table 6; Figure 3D).

## Discussion

Based on the current situation of a high incidence of chronic respiratory diseases and strained medical resources, this study systematically evaluated the effects of nurse-led aerobic pulmonary rehabilitation in patients with moderate to severe COPD. The results showed that this model had significant advantages in improving patients’ exercise tolerance, quality of life, and physiological status, suggesting that nurse-led rehabilitation interventions can not only enhance patients’ functional recovery but also achieve more efficient and sustainable rehabilitation management under resource-limited conditions.

The nurse-led pulmonary rehabilitation model constructed in this study demonstrates unique clinical value by fully leveraging the coordinating role of nursing staff in multidisciplinary collaboration and the advantages of whole-process management (19). It should be noted that the ability of nurses to undertake the core implementation and coordination tasks described in this study was based on their receipt of systematic, specialized training. After training, nurses were required to pass both theoretical and practical competency assessments before they could independently perform the intervention. During the intervention period, they underwent on-site quality checks by the rehabilitation specialist once every 2 weeks. Therefore, the nurse-led model presented in this study is built upon a clear framework of qualification and quality supervision, rather than simply delegating complex tasks to insufficiently trained nursing staff. In this model, the pulmonary rehabilitation nurse assumes the core coordinating function of a case manager within the multidisciplinary team, responsible for implementing the rehabilitation plan, monitoring patient progress, facilitating communication among team members, and ensuring continuity of care from admission to discharge follow-up. Clinical decisions regarding medical management remain under the authority of the respiratory physician, and exercise prescriptions are approved by the rehabilitation specialist (20). Nurses, under the supervision of the respiratory physician and following the exercise prescription protocols established by the rehabilitation specialist, dynamically adjust the intensity and frequency of breathing training and fluid management based on daily patient assessments. Any adjustment beyond the prespecified protocol requires approval from the rehabilitation specialist or respiratory physician (15). This real-time feedback and dynamic optimization mechanism keeps the rehabilitation plan in a state of continuous individualized adjustment, maximizing rehabilitation benefits while ensuring safety (15). At the same time, this model achieves a deep integration of professional techniques and humanistic care (16). Peer support and psychological counseling are organically integrated into the rehabilitation process, and semi-structured interviews are used to analyze the root causes of patients’ negative emotions, creating a positive rehabilitation atmosphere (21). This holistic care model, which emphasizes both physical and mental health, not only alleviates patients’ anxiety and depression but also enhances long-term treatment adherence by boosting self-efficacy (21). From the perspective of resource allocation, the coordination of respiratory therapists, rehabilitation physicians, nutritionists, and other roles by the pulmonary rehabilitation nurse significantly improves team operational efficiency (15). Nurses are responsible for summarizing and analyzing daily intake and output data, assessing fluid balance status in conjunction with indicators such as blood pressure and central venous pressure, and promptly communicating with the respiratory physician to propose adjustments when issues are identified (16). This not only reduces the workload of physicians but also ensures timely responses to problems. Against the backdrop of strained nursing human resources, this model achieves seamless multidisciplinary intervention by clarifying the coordinating role of nurses, avoiding rehabilitation interruptions or redundant work caused by poor communication in traditional models, and providing a practical pathway for promoting sustainable pulmonary rehabilitation services under resource-limited conditions.

Research data showed that in both the ITT and PP populations, the 6MWD in the intervention group at 4 weeks was significantly better than that in the control group, indicating that the nurse-led pulmonary rehabilitation model has a significant advantage in improving exercise tolerance in patients with moderate to severe COPD, and the dropout was random. The potential mechanisms underlying these findings remain speculative and require further investigation. Based on our observations and previous literature, we hypothesize that several factors may have contributed to the observed improvements. First, the nurse, acting as the whole-process manager, dynamically adjusted the exercise intensity based on daily vital signs and subjective feelings, which may have ensured a better match between training intensity and the patient’s real-time status (22). However, this interpretation is hypothesis-generating and should be confirmed in future studies using process evaluation or mechanistic biomarkers. For example, during progressive exercise training, the nurse decided whether to initiate or terminate training based on muscle strength assessment and oxygenation status, effectively avoiding the risk of fatigue or hypoxia caused by inappropriate exercise, thereby ensuring the safety and sustainability of the training (22). This model integrated airway clearance techniques with respiratory muscle training, improving patients’ ventilation efficiency and respiratory muscle endurance through phased guidance on diaphragm activation, resistance training, and breathing pattern reconstruction, laying a physiological foundation for the improvement of exercise tolerance (23). The nurse-led motivational psychological support significantly enhanced patients’ self-efficacy and treatment adherence, enabling them to participate more actively in daily training, which cumulatively led to substantial improvements in exercise capacity (21). Regular aerobic exercise directly promoted the increase in 6MWD by improving skeletal muscle oxidase activity, reducing systemic inflammatory responses, and enhancing cardiopulmonary efficiency (24). Meanwhile, the precise fluid management and nutritional support under the nurse-led model further optimized patients’ circulatory function and energy metabolism, providing systemic support for the improvement of exercise performance (15). Through the synergistic effects of individualized adjustment, technical integration, and psychological motivation, nurse-led pulmonary rehabilitation promoted the enhancement of patients’ exercise tolerance via multiple pathways, including functional training, physiological adaptation, and behavioral change.

In this study, the nurse-led pulmonary rehabilitation model also played a positive role in improving the quality of life of patients with moderate to severe COPD. As case managers, nurses dynamically assessed and adjusted interventions in real time, integrating airway clearance techniques, respiratory muscle training, and progressive exercise training to directly alleviate patients’ dyspnea symptoms and enhance exercise tolerance, thereby reducing the life distress caused by limitations in daily activities (25). This model emphasized early oral feeding and precise fluid management, combined with traditional Chinese medicine abdominal acupoint massage and hot herbal pack application, which not only optimized patients’ nutritional status and circulatory function but also relieved physical discomfort by harmonizing Yin and Yang and warming the meridians to unblock collaterals, indirectly improving life comfort (26). At the same time, regular pulmonary rehabilitation directly alleviated dyspnea and fatigue in COPD patients by reducing systemic inflammatory responses, improving cardiopulmonary efficiency, and enhancing skeletal muscle function (27). The continuous health education and behavioral guidance provided under the nurse-led model further promoted patients’ self-management abilities, enabling them to better cope with the physiological and psychological challenges posed by the disease, ultimately reflected in the substantial decline in CAT and SGRQ scores.

Nurse-led pulmonary rehabilitation also led to significant improvements in patients’ physiological status, as evidenced by marked increases in both the BI and Borg scores. The progressive exercise training implemented in this model encompassed multiple stages, including bed-based, sitting, standing, and walking activities, with nurses determining training intensity and duration based on daily muscle strength assessments and oxygenation status (15). This individualized adjustment not only avoids exercise-related adverse events but also promotes the enhancement of skeletal muscle oxidative enzyme activity and muscle strength through regular stimulation, directly reflected in the improvement of activities of daily living as measured by the BI (28). The active cycle of breathing techniques and cough suppression training, combined with phased respiratory muscle resistance training, significantly enhanced patients’ spontaneous sputum clearance ability and respiratory muscle endurance while reducing the degree of dyspnea, which was reflected in the optimized Borg scores (10). Research indicates that nurse-led health education and psychological support can enhance patients’ self-efficacy and treatment adherence, motivating them to participate more actively in daily training (16). This behavioral change, superimposed on the effects of physiological training, jointly promoted improvements in neuromuscular coordination and exercise tolerance, which may be the primary mechanism underlying the enhancement of patients’ physiological status indicators (29). It is evident that through the synergistic effects of circulatory optimization, muscle training, airway clearance, and nutritional support, nurse-led pulmonary rehabilitation comprehensively promoted patients’ physiological status via multiple pathways, including cardiopulmonary function, physical activity capacity, and subjective perception of fatigue.

However, this study has certain limitations. First, as a single-center study with a relatively limited sample size, future research should expand the sample to enhance the extrapolation and generalizability of the findings. Second, the intervention period was only 4 weeks; although significant improvements were observed in multiple indicators, the long-term maintenance effects of the nurse-led pulmonary rehabilitation model could not be assessed, necessitating extended follow-up evaluations to determine the persistence of rehabilitation benefits. In addition, this study has a short follow-up duration, a single-center design, potential performance bias due to the open-label nature, and a limited description of nurse training and competency assessment. These factors may affect the generalizability and reproducibility of the findings. Finally, as an exploratory study, some mechanistic explanations remain at the inferential level. Future research should incorporate blood biomarker detection or imaging assessments to further elucidate the biological mechanisms through which the nurse-led pulmonary rehabilitation model improves patient outcomes.

## Conclusion

In this single-center, 4-week trial of 80 patients with moderate to severe COPD, the nurse-led aerobic pulmonary rehabilitation model was associated with improvements in exercise tolerance, quality of life, and physiological status compared with routine care. These preliminary findings suggest that this model may offer a feasible alternative for pulmonary rehabilitation in resource-limited settings where access to physiotherapists is restricted. However, given the short intervention period and modest sample size, further research with larger samples, longer follow-up, and multicenter designs is needed to confirm the effectiveness, sustainability, and generalizability of this approach.

## Data Availability

The raw data supporting the conclusions of this article will be made available by the authors, without undue reservation.
